# Experiential Learning in a Biomedical Device Engineering Course: Proposal Development and Raw Research Data-Based Assignments

**DOI:** 10.1007/s43683-022-00094-z

**Published:** 2022-12-07

**Authors:** Noah Goshi, Gregory Girardi, Hyehyun Kim, Erkin Seker

**Affiliations:** 1 Department of Biomedical Engineering, University of California-Davis, Davis, CA 95616 USA; 2 Department of Electrical and Computer Engineering, University of California-Davis, Davis, CA 95616 USA

**Keywords:** Device engineering, Proposal development, Peer review, Concept inventory, Remote instruction

## Abstract

There is a need for novel teaching approaches to train biomedical engineers that are conversant across disciplines and have the technical skills to address interdisciplinary scientific and technological challenges. Here, we describe a graduate-level miniaturized biomedical device engineering course that has been taught over the last decade in in-person, remote, and hybrid formats. The course employs experiential learning components, including a proposal development and review that mimic the National Institutes of Health process and technical assignments that use raw research data to simulate a research experience. The effectiveness of the course was measured via pre-/post-course concept inventory surveys as well as course evaluations with targeted questions on the learning instruments. Statistical comparison of pre-/post-course survey scores suggests that the course was effective in students achieving the learning objectives, and comparison of relative increase in pre-/post-course survey scores across different instruction formats (i.e., in-person, remote, hybrid) showed minimal difference, suggesting that the teaching elements are readily transferrable to remote instruction.

**Supplementary Information:**

The online version contains supplementary material available at 10.1007/s43683-022-00094-z.

## Challenge Statement

Many exciting scientific and technological challenges require a multidisciplinary approach and scientists/engineers who can communicate across core disciplines. These numerous multidisciplinary topics range from biomedical device engineering to sustainable energy production. In order to train a workforce to tackle these diverse challenges, there is a need for thematic courses that expose students to essential knowledge and tools to facilitate the pursuit of a specific idea with a multidisciplinary scope.^7^ An emerging challenge is how to structure such a course to encourage student engagement on diverse disciplinary topics while leveraging their core department’s foundation of knowledge. Biomedical engineering with its inherent interdisciplinary nature and ever-expanding knowledge base embodies many of these educational challenges.^7,8^ For example, in order for students to engineer miniaturized biomedical devices, they need to develop an understanding of microfabrication, nanotechnology, surface science, basic biological principles, and sensor/actuator operation principles. Therefore, there is a need for pedagogical approaches to effectively train students on both the foundational concepts and their real-life relevance. In addition, these teaching approaches should ideally be transferrable to the remote instruction format, as remote instruction during the pandemic has accelerated the development of online and/or hybrid courses in many institutions.^6^

## Novel Initiative

In order to address the challenge of effectively training students interested in biomedical device engineering, Seker developed and taught a graduate-level course titled “Micro- and Nano-Technology in Life Sciences” with contributions from several teaching assistants. The course collectively utilizes lectures, technical assignments, and the development of a fellowship proposal as a framework inspired by the Revised Bloom’s Taxonomy^1^ and active learning pedagogy^4^ to effectively teach a highly-interdisciplinary biomedical device engineering course and instill critical thinking skills. This article specifically focuses on the experiential elements, including (i) the proposal development-centered approach, and (ii) technical assignments that employ raw research data. In summary, the proposal development process mimics a National Institutes of Health (NIH) proposal submission and review process and teaches the essential skill of deconstructing an idea into achievable and measurable research tasks. One intention of the proposal-related exercises is to assist the students in exploring research topics of interest and learn how to formulate a research plan and identify the gaps in their technical knowledge. Peer-review of proposals by the students prompts them to comprehend an unfamiliar scientific topic well enough to provide academically-sound criticism. The technical assignments, on the other hand, complement the proposal assignments by focusing on practical research tools and techniques (image processing, statistical analysis) to analyze raw research data from the instructor’s laboratory. Taken together, the proposal development and technical skills acquired in the course prepare the students for embarking on the interdisciplinary field of biomedical device engineering (to be discussed later with reference to student evaluations).

## Course Components

### General Structure

The course has been offered annually since 2012 with the two instances (2020 and 2021) delivered via remote instruction. The class (4-unit course) meets twice a week over ten weeks, which is the standard duration of an academic quarter at University of California, Davis. The course enrollment grew over the years from ~12 students to 40+ students. The students are generally MS and PhD students in their first two years as well as a few senior undergraduate students. The students have evenly represented programs in electrical and computer engineering, biomedical engineering, mechanical and aerospace engineering, materials science, and chemical engineering.

The course begins with a “big-picture” lecture on the history of miniature biomedical devices and an outline of enabling disciplinary topics. Through the duration of the course, the students receive didactic training through discussion-based lectures on microfabrication, surface chemistry, basic biological principles, and survey of miniature biomedical devices, as outlined in Table 1. Technical assignments complement the lectures and evaluate a student’s mastery of the topics while teaching techniques that can be used in their own research projects (see Supporting Information for example assignments). Prior to the pandemic-related restrictions, the last week of the course consisted of laboratory demonstrations of surface modification, microfluidic devices, cell culture, and microscopy to exemplify some of the key concepts introduced in the lectures as well as the tools/processes that generated the raw data used in technical assignments.

**Table 1:** Lecture content and assignment schedule.

Lecture	Lecture content	Assignments
1	Introduction & Course outline	Proposal assignment (Topic identification)
2	Surface science	
3	Mass transfer	Technical assignment 1
4	Anatomy & Physiology	
5	Pathology	Technical assignment 2
6	Microfabrication	
7	Microfabrication	Technical assignment 3
8	Nanofabrication	
9	Characterization	Technical assignment 4
10	Grant proposal	Proposal assignment (specific aims page)
11	Materials	
12	Packaging	Midterm examination
13	Sensors	Proposal assignment (peer-review)
14	Actuators	
15	Biological models	Assignment 5
16	Biointerface	Proposal assignment (final proposal + rebuttal)
17	Big picture	Proposal assignment (elevator pitch presentation)

For the midterm examination the students had to design a microfluidic electrochemical biosensor and include a detailed discussion of the microfabrication steps, enabling fluidic principles, bio-functionalization, packaging, and process compatibility. Except for the midterm, the students are encouraged to collaborate on assignments, as with the diverse disciplinary background each student had a different strength (e.g., microfabrication, biology, surface chemistry). This modeled “team science” at a classroom scale, which will serve as a vital tool to succeed in today’s highly interdisciplinary scientific environment.^2^ During remote instruction due to the pandemic, the take-home midterm focused on COVID diagnostics, where the students were asked to employ the course material (microfabrication, biology, etc.) to develop an electrochemical sensor for SARS-CoV-2 detection in biological samples. Overall, the diverse yet synergistic learning instruments in the course maintained student engagement throughout the duration of the course (as supported by student comments/evaluations to be discussed later).

### Proposal Development

To inform the proposal development process, we use successive assignments with detailed instructions (see Supporting Information) to assist students in systematically constructing proposal components (e.g., specific aims, research approach), conducting peer reviews, and composing a response to reviewers as a part of the final revised proposal, where complementary approaches have been explored for instruction on manuscript development and peer review.^5^ At the beginning of the course, students begin to identify a research question through directed readings and consultation with the instructor. The students submit a list of three potential topics that need to be at the intersection of miniaturized device fabrication and its application to a biomedical need/question. As part of subsequent assignments, students prepare a NIH-style specific aims page and, based on the instructor’s feedback, are given two weeks to write a three-page short proposal built on the NIH proposal structure (i.e., Significance, Innovation, Approach). With the goal of exposing students to the peer review process, each student reviews two of their classmates’ proposals and fills out an online Google Forms-based score sheet and provide comments on categories adopted from the NIH reviews (e.g., significance, innovation, approach). The critiques and scores from the peer reviewers and the instructor are compiled into a summary statement-like document and forwarded to each student. As the final proposal-related assignment, the students write a half-page response to reviewers’ comments and revise the final proposal accordingly. In addition, each student delivers an elevator pitch-style presentation on their proposal.

The proposal constitutes a working draft for a fellowship application to internal or external funding sources, as well as for doctoral proposal exams. This builds the motivation to create an end-product with a potential for prestige, academic utility, and monetary value. The critical thinking and writing skills developed through the fellowship proposal activities can be broadly transferrable to non-academic proposals, such as business plans, thereby making the course relevant to various career paths. Overall, this framework enables an experiential learning environment, where students are motivated by the practicality and real-life similarity of the proposal process and engaged in lectures and assignments to more effectively strengthen their technical knowledgebase (as evidenced by the *Concept Inventory Survey* assessment and targeted course evaluation questions discussed in the final section).

### Technical Assignments

The technical assignments (outlined in Table 2 and illustrated in Supporting Information) have the overarching goal of balancing practical relevance and theory.^9^ For example, the course introduces ImageJ, a commonly-used image processing software, which the students use to complete several technical assignments. Students use this software package to analyze scanning electron microscopy images of biomedical device coatings and make theoretical calculations on drug loading capacity of a biomedical implant coating. In other assignments, students develop a microfabrication process to engineer a miniature diagnostic ultrasonic transducer and statistically analyze epifluorescence images of cells grown on drug-eluting nanoporous coatings loaded with different concentrations of anti-mitotic pharmaceuticals. Finally, another assignment focuses on analysis of UV–Vis absorbance spectroscopy data from chromophores with different concentrations with the goal of creating calibration curves—a pillar of bioanalytical chemistry. The common theme in each assignment is its strong connection to raw experimental data and existing biomedical devices. Data analysis and interpretation of fresh data with not-yet-known conclusions create a real research feel for the students and enhance their engagement, thereby constituting an experiential learning environment. The technical assignments and proposal-related assignments are interlaced throughout the course, as shown in Table 1.

**Table 2:** List of technical assignments related to the core concepts.

Assignment	Description
1	Standard curve generation from raw UV-Vis absorbance spectra of food coloring
2	Nucleic acid sequence to amino acid sequence translation; physicochemical properties of the protein
3	Microfabrication process development for a MEMS-based ultrasonic transducer
4	Image processing of porous medical device coatings to estimate drug loading capacity
5	Image processing of cells treated with anti-mitotic drugs for statistical comparison of cell numbers

## Reflection

In order to evaluate course effectiveness, we employed a concept inventory-based assessment approach^3^ and added targeted questions on course evaluations. During the first and last class of the quarter, we administered a survey of nine questions (Table 3) to evaluate students’ conceptual interdisciplinary knowledge of biomedical device engineering. Each question was scored as 0 (incorrect), 0.5 (partially correct), or 1 (correct) by the instructor in all course offerings to maintain uniformity in scoring and the factual nature of the questions were intended to minimize subjective bias in scoring. Questions with two distinct sub-questions were scored as 0.5 points each. The scores from the first and last classes are referred to as “pre” and “post”. The questions tested basic knowledge of relevant topics, including microfabrication, biology, statistics, fluid mechanics, nanotechnology, and bioanalytical techniques.

**Table 3:** Concept inventory survey.

Question	Concept question
1	Outline a microfabrication process to create 500 μm-wide 1 μm-thick aluminum traces on a glass substrate.
2	How can you bond a silicone elastomer to a glass surface?
3	What is Reynold’s number?
4	What is the difference between convective vs. diffusive mixing? Which one is more likely in micro-channels?
5	What chemical molecules would you attach (and how) on a gold surface to make it hydrophobic or hydrophilic?
6	What are the four main biomolecular building blocks that form biological systems (e.g., amino acid)?
7	What is the central dogma of biology?
8	What are the functions of primary and secondary antibodies in immunostaining?
9	What statistical test would you use to compare two normally-distributed groups? How about more than two groups?

**Figure 1:**
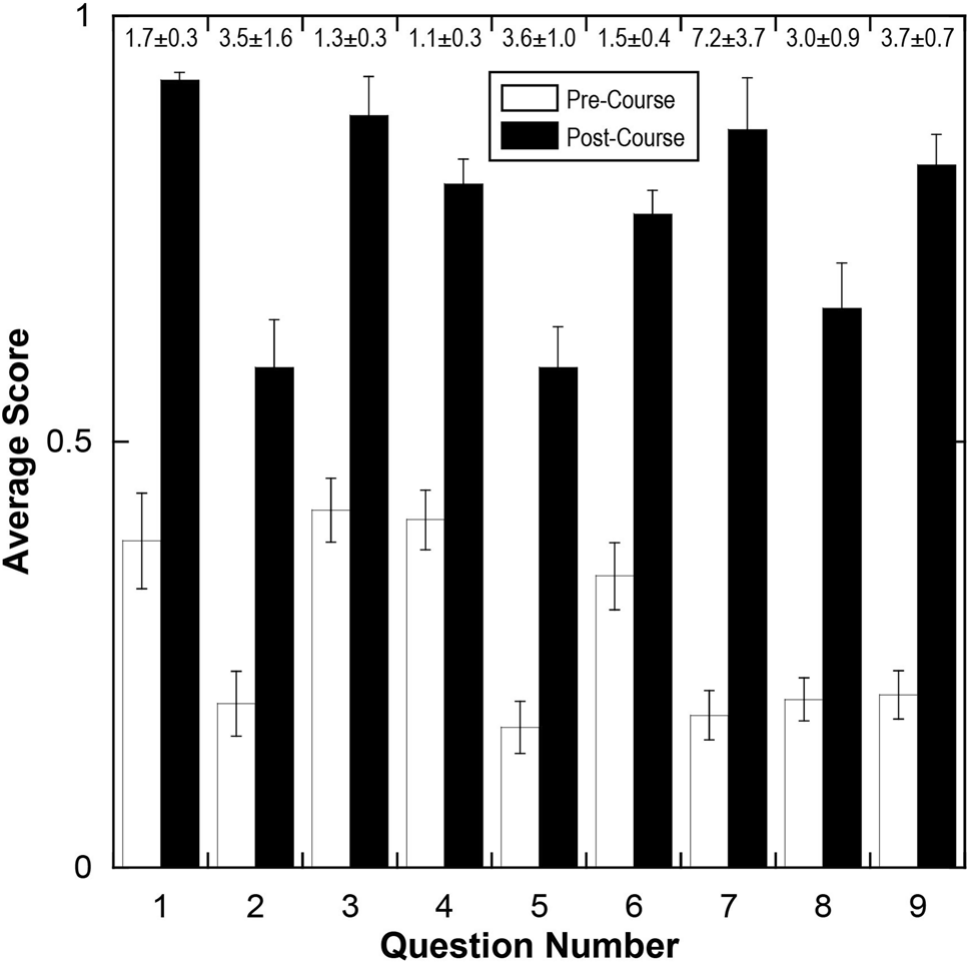
Summary of average scores for each question on the concept survey (Table 3) pooled with the responses from the courses taught 2015 through 2022. Each question was scored as 0 (incorrect), 0.5 (partially correct), or 1 (correct). The error bars indicate standard error with *n* = 72 for pre-course and post-course. The statistical comparison of pre- and post-course average scores via a Mann-Whitney test yielded a *p*-value of at least 0.001 for each question. The average ± standard error annotations above the columns show the relative increases in average score ([post – pre] / pre)] for each question.

As a measure of how well the students achieved the learning outcomes for each concept (Table 3), we calculated the question-wise relative increase in average score (*[post – pre] / pre*) for each cohort (e.g., Figure 1 shows pooled data for all cohorts). Note that the number of students that formed the average pre- and post-course scores per question was 188 and 113 respondents respectively. In order to test whether the instruction format had an influence on students’ learning, we clustered the cohorts based on their instruction format: in-person (2015–2019; *n* = 45), remote (2020–2021; *n* = 18), and hybrid (2022; *n* = 9). We then compared three groups (with respect to cohort-wise pooled relative score increases) with a Kruskal-Wallis one-way ANOVA test, which resulted in a *p*-value of 0.89 (with chi-squared approximation), indicating minimal difference between the three groups. Justified by this, we pooled the average scores from all the cohorts and compared the pre- and post-average scores question-wise with a Mann-Whitney test, and found a significant difference between pre- and post-course average scores (*p* < 0.001 for each question [*n* = 8] and *p* < 0.0001 for all questions pooled [*n* = 72]) indicating a significant increase in the overall understanding of the core concepts. Figure 1 illustrates the average scores (pre-course and post-course) pooled for all cohorts on a question-by-question basis. In summary, the instruction format worked equally well as in-person and remote and the students demonstrated a significant increase in their understanding of the core concepts.

As a complementary measure of the course effectiveness, the student evaluations of the course from annual offerings between 2016 and 2022 are shown in Table 4. Overall, the students found the education value of the course high (4.6 ± 0.7; *n* = 141) and indicated that their interdisciplinary knowledge on the design of biomedical devices increased (4.6 ± 0.6; *n* = 136). The course also contributed to increasing confidence in proposal development (4.4 ± 0.7; *n* = 103) and developing an interest in biomedical device engineering (4.3 ± 0.8; *n* = 101). An additional specific question (used in 2022) related to the value of the experiential learning components indicated that the students gained a sense of research in the biomedical device engineering area (4.2 ± 1.1; *n* = 30).

**Table 4:** Course evaluation questions (2016-2022).

Course Evaluation Question	Mean	**SD**	N
Please indicate the overall educational value of the course *(excellent | very good | satisfactory | fair | poor)*	4.6	0.7	141
My interdisciplinary knowledge of the concepts that constitute the design of biomedical devices (e.g., microfabrication, surface science, essential biology, and/or biomedical devices) increased as a result of taking this course *(5: Strongly agree - 1: Strongly disagree)*	4.6	0.6	136
My confidence in writing a grant proposal (e.g., fellowship application) increased as a result of the proposal-related assignments of this course *(5: Strongly agree - 1: Strongly disagree)*	4.3	0.8	136
I became more interested in biomedical device engineering as a result of taking this class *(5: Strongly agree - 1: Strongly disagree)*	4.3	0.9	134
Experiential learning components (e.g., real research data used in practical technical assignments, grant proposal assignments mimicking the actual federal grant proposal process) assisted me in getting a sense of research in the biomedical device engineering area. **[Only for 2022 course offering]** *(5: Strongly agree - 1: Strongly disagree)*	4.2	1.1	30

Overall, the student evaluation comments centered around enhanced student interest to apply miniaturization technology to health care and the usefulness of the proposal component complement the quantitative findings:

*“The biggest takeaway from this class was the grant proposal practice. I wish this was one of the first things I learned once I got into grad school. This exposure to the grant proposal writing process was very insightful and needed.”*

*“This was quite an insightful course, especially the introduction to grant proposal writing.”*

*“The breadth of the course serves as an excellent introduction to a range of topics in microfabrication for biomedical applications.”*

*“…really good course and help 1st or 2nd year students finding their directions.”*

*“The assignments gave a very practical view to the learned concepts.”*

The course components collectively were in line with the Revised Bloom’s Taxonomy^4^, where (i) the technical assignments prompted the students to “remember, understand, apply” the fundamental knowledge from the lectures to solve problems based on raw research data; (ii) the design-based midterm and the proposal development required the students to “analyze” the raw data and “apply” their knowledge and “create” original work (e.g., novel device design); and (iii) the peer-review of proposals provided the opportunity to “evaluate” others’ work and knowledge (e.g., proposal peer-review rubric). The course format and the experiential learning components described in this article should be readily applicable to different courses. To that end, the instructor uses this structure in other courses, including a graduate-level introductory neuroengineering course. The most significant challenge is the scalability of the proposal-related assignments. While the technical assignments can be reviewed by the teaching assistants, the proposals on a variety of different topics require the instructor’s evaluation. This becomes significantly more time-consuming for large classes (e.g., over 25 students). A possible addition to the proposal component may involve creating breakout peer-review groups that simulate the collective discussion environment of a study section. In general, the course (specifically the proposal development component and technical assignments) has been successful. A potential implementation of the proposal component could be a collaboration on teaching a proposal-driven course between two or more universities. In this scenario, the students from different institutions participate in writing, reviewing, and revising each other’s proposals and consequently develop a working knowledge of an interdisciplinary field aided by proposal-centered activities.

## Supplementary Information

Below is the link to the electronic supplementary material.

Supplementary file1 (DOCX 946 kb)

## Data Availability

Examples of technical and proposal-related assignments are included as Supporting Information. Midterm examination and additional tools are available upon request.

## Code Availability

The ImageJ codes are available upon request.
