# Supplementary material for: Experiential Learning in a Biomedical Device Engineering Course: Proposal Development and Raw Research Data-Based Assignments
Source: Biomed Eng Educ. 2022 Dec 7;3(2):201–7. doi: 10.1007/s43683-022-00094-z (PMC9734624; doi:10.1007/s43683-022-00094-z)
Supplement: Supplementary file 1 — Supplementary file1 (DOCX 946 kb) [file 43683_2022_94_MOESM1_ESM.docx]

***Supporting Information for***

**Experiential Learning in a Biomedical Device Engineering Course:**

**Proposal Development and Raw Research Data-Based Assignments**

Noah Goshi^1^, Gregory Girardi^1^, Hyehyun Kim^1^, Erkin Seker^2,*^

^1^Department of Biomedical Engineering, ^2^Department of Electrical and Computer Engineering, University of California- Davis, Davis, CA 95618, USA

**Examples of Technical Assignments**

Assignment 1

*This assignment involved the use of absorbance data collected in the laboratory to teach how to create standard/calibration curves.*

UV-Vis (absorbance) measurements are the workhorse of bioanalytical chemistry laboratories. See the Excel sheet “Absorbance.xls” under “Files/Assignments” on Canvas. The worksheet includes the absorbance data for a common food coloring dye “Erioglaucine” at various concentrations in an aqueous solution. Three measurements (adjacent data columns) were taken for each concentration.

- 1. Superimpose the average absorbance profile (absorbance vs. wavelength) of the three measurements for each concentration
  2. In the plot, use error bars to display standard deviation of the three measurements for each concentration
  3. Comment on the absorbance profile – how many peaks are there?
  4. Create standard (calibration) curves (absorbance vs. concentration) based on the data – use as many features of the absorbance profile as possible (hint: there are at least three possible standard curves that can be produces) and briefly describe your approach to constructing the standard curves
  5. Determine curve-fitting equations that describe each standard curve
  6. Determine the R^2^-value for each curve-fit – do the values differ between the standard curves?
  7. What absorbance value(s) would a concentration of 0.5 mM erioglaucine correspond to?

Assignment 3

*This assignment focused on employing the microfabrication techniques discussed in the lectures to develop a fabrication process for a miniaturized biomedical device. In addition, the students downloaded ImageJ to familiarize themselves with the software for the future assignments.*

1. Below are the side and top views of an electrostatically-actuated membrane. When a DC bias and superimposed sinusoidal voltage are applied to the bottom and top electrodes, membranes can produce ultrasonic pressure waves and even detect reflected pressure waves. This can be used for non-invasive medical diagnostic platforms, as well as non-destructive material testing.


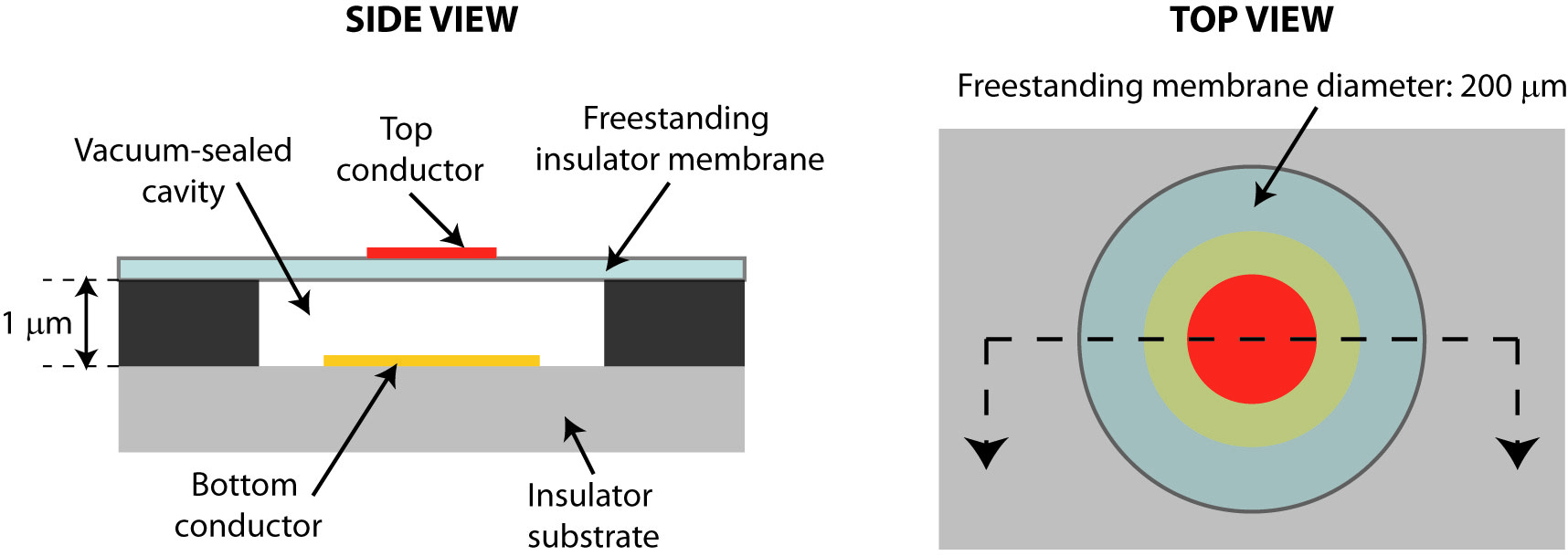


- 1. Develop a microfabrication process to produce these devices (describe what deposition, etching, and patterning methods you would use)
  2. Draw the process flow using both side and top views of the device
  3. Draw each photo-mask with alignment marks used for the process (features for a single device is sufficient, but the polarity of the mask should be consistent with your process)

IMPORTANT NOTES:

- The colors in the image above are different for clarity – it does not necessarily mean different colors represent different materials or vice versa. The relative vertical (layer thicknesses) and horizontal (feature geometry on masks) dimensions are consistent within themselves.
- The cavity between the membrane and the substrate should be vacuum-sealed.
- Pick your process conditions (e.g., deposition method, etc.) by paying attention to the dimensions
- Use “Microfab Etch Rates I and II” reference publications under *Reading* folder to evaluate the compatibility of each process with each other (to the best of your knowledge)

1. ImageJ software (We will be using this software for analyzing nanostructures and biological activity):
   1. Install the software (<http://fiji.sc/wiki/index.php/Fiji>)
   2. Familiarize yourself with opening images: (<http://fiji.sc/wiki/index.php/Getting_started>)

Assignment 4

*This assignment focused on using ImageJ to analyze scanning electron microscope images. In the second part of the question, the students applied the image processing techniques to estimate the surface area and surface-bound drug loading.*

1. Thresholding (also known as *segmentation*) is an essential part of image processing. There has been considerable research in developing segmentation algorithms – a few of them are included as plug-ins in ImageJ (Image/Adjust/Auto Threshold). The objective of this exercise is to perform particle analysis using one automatic algorithm (pick one that produces a reasonable representation of the void structure and use the same set for each image) and one manual thresholding (Image/Adjust/Threshold). Use the same “Particle Analysis” parameters for all analyses. Run *median filter* with radius=2 pixels after thresholding. Count all particles with sizes between zero and infinity. Use the SEM images in folder “HW4-Q1” under Assignments folder.
   1. Tabulate the following for each analysis:
      1. Thresholding method (it may be a good idea to try all and pick one)
      2. Threshold value
      3. Number of voids
      4. Average void area
      5. Void ratio (i.e., percent footprint covered by the particles)
      6. Histogram illustrating the distribution of void areas for each image
      7. Compare the results (iii, iv, v) for the 2 different segmentation methods (manual and auto)
   2. Discuss how the results from each segmentation algorithm differ and if this difference is consistent across the two images. Briefly describe the operation principle of the algorithm that you used and cite reference.
2. Image analysis is a powerful tool to extract certain device properties, such as overall surface area and volume of pores in a material. The objective of this exercise is to estimate the total surface area (including internal pore surfaces) of a biomedical device coating. Use the image in folder “HW4-Q2”. This is the SEM image of a porous silica glass with through-pores (image size: 13972 nm x 11178 nm). The film thickness is 1 micron.
   1. Answer i through iv of question 1:part (a) for the image in this question
   2. Include a copy of the thresholded image
   3. If we were to coat a spherical (radius=3 mm) prosthetic implant surface with this film (1 micron thickness), how much would the total surface of the implant increase compared to a non-coated planar surface?
   4. If we were to soak the porous implant surface with an anti-inflammatory drug solution of dexamethasone to ensure complete coverage of the available surface with a monolayer of the drug at highest molecular packing density possible, how many moles of dexamethasone would be surface-loaded onto the implant? State all your assumptions.

NOTE: ImageJ allows you to record/run macros. Feel free to use them for repetitive analyses and include the macro script with your homework.

Assignment 5

*This assignment continued to build on the use of ImageJ and this time applied it to analyzing epifluorescence images of immunostained cells that went under anti-mitotic drug treatment.*

1. Statistical comparison of cell morphology/number in response to a certain treatment (e.g., drug, radiation, a new material, etc.) is a typical biological experiment. You are asked to evaluate the effect of an experimental drug on astrocytes (supportive brain cells). You use three groups of cell cultures: (i) *Control Group*, no drug treatment; (ii) *5X Group*, where cells are treated with 5X concentration of the drug; and (iii) *50X Group*, where cells are treated with 50X concentration of the drug. After the cells are exposed (or not) to the drug for one day, they are stained (see below) and imaged. Image files for each group are under “Homework 6” folder on Canvas.
   1. Conduct image analysis to quantify the ***number of cells*** and ***average nucleus size*** in each image. (Tip: Using macros can significantly reduce the analysis time)
   2. Tabulate the number of cells and average nucleus size for each image.
   3. Tabulate the average and standard deviations of the measurements for each group (i.e., Control, 5X, 50X).
   4. Plot the averages and standard deviations (error bars) of each group using a column graph.
   5. Perform a single-factor ANOVA (analysis of variance) to test whether the three groups are statistically the same (p-value > 0.05).
   6. Perform two-tailed Student’s t-tests to compare the effect of drug treatment on the number of cells and average nucleus size – record p-values for each comparison.
   7. Interpret your results with particular emphasis on whether there is (or not) a statistical significance – how does drug treatment affect number of cells and average nucleus size?


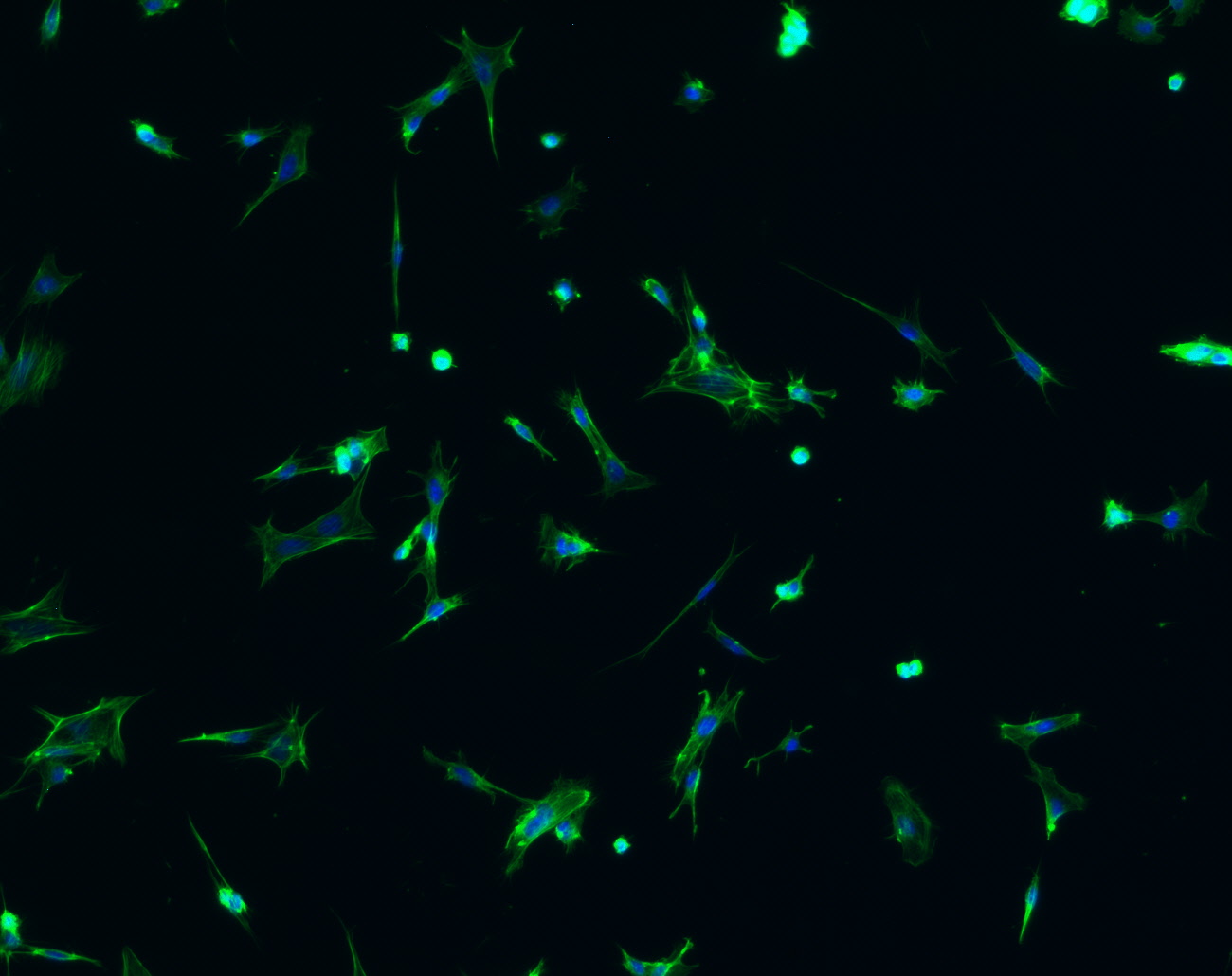


**Figure.** Blue stain is DAPI, which marks cell nucleus; green stain is phalloidin-Alexa 488, which marks cell cytoskeleton.

**Examples of Proposal and Presentation Assignments**

*The series of assignments below are given throughout the quarter to allow students to gradually build their proposals, carry out peer review, and revise their proposals based on the feedback from two peers and the instructor. The students also present their proposal in the form of an elevator pitch and receive feedback with a similar peer review process.*

Assignment 1: Literature Review & Topic Identification

The assignment is to conduct a literature review and identify 3 topics that you would want to learn more about and consider writing a proposal on. The topics should be at the intersection of micro-/nano-technology and biology/medicine. Please see Reading/Proposal sub-folder for an example proposal from previous years.

Submit your assignment as a pdf document with a 3-5 sentence description of each topic and citations to the relevant references. The objective of the assignment is to get you thinking about the proposal early on in the course. This assignment will only be graded as "Complete" or "Incomplete", with a reasonable effort earning a "Complete" grade.

Assignment 2: Specific Aims

The Specific Aims page is due on [DATE] on Canvas. This will be the core of your proposal and should have a biomedical/biological focus, that is, topics related (but not limited) to technology development for studying a biological phenomenon or diagnosing/treating disorder. In the final proposal, I would like to see some basic biological experiments proposed. I provided feedback on your first assignment related to Topic Identification. While constructing the aims, you should pay special attention to having well-defined experimental parameters to vary and experimental outcomes to measure. In other words, while the proposal does not need to have high innovation, it should have a logical and systematic experimental structure.

**The page margins should be 1 inch all around and the font should be 11 pt Arial single spaced. It should be exactly 1-page long, have a title, and have two aims.** It is not necessary to include citations to literature references in the Specific Aims page.  The structure should follow what is described in "Specific Aim Development" document posted under Reading/Proposal on Canvas. Feel free to look at proposal example from the previous years on Canvas. I will also enable a discussion board on proposals so that you can interact among yourselves. Please feel free to reach out to me for technical advice and general proposal-related questions.

Use the proposal assignments to dwell deeper into bioengineering topic that you are interested in. Good luck!

Assignment 3: First Proposal

The first submission of the proposal (based on your specific aims) will be due on [DATE]. Please make sure that you submit it by the deadline, as I will assign proposals for peer review right away. **I just submitted comments on your Specific Aims.** **Please read my suggestions carefully and take them into account in your first proposal submission.**I also strongly encourage you to review the structures of the example proposals from previous years (on Canvas).

The proposal should include the following:
- 1 page (exact length) revised Specific Aims (based on individual comments you received)
- 3 pages (exact length) Proposal Body (excluding Specific Aims page and References) 
- 1 page (max length) References
- Everything should be written with 11 pt Arial (figure captions can be 10 pt); 1-inch margins all around

More on the Proposal Body:
The write-up should be divided into the three sections below:
- Significance (~0.75 page): (i) State the importance of the proposal; (ii) summarize key literature that is relevant to what you are proposing; and (iii) state the problems/gaps that you propose to address.
- Innovation (~0.25 page): State what your proposed solution is to the stated problems. I understand that it is hard to make this completely novel, but try your best.
- Approach (~2 pages): (i) Discuss the experiments/analysis you propose to do for each Specific Aim (two total); (ii) include a brief timeline for experiments; and (iii) state expected results, potential issues/alternative approaches, and future directions.

Use figures/charts/graphs as necessary but not to fill in space. You probably won't have your own preliminary data, but feel free to adopt key findings from literature (with citations) to rationalize the methods you propose to use.

I will circulate proposals between you for peer-feedback, which you will have the opportunity to use for revising the proposal for final submission during the finals week. I am pasting below the review criteria so that you can ensure the necessary points are addressed in your proposal.

Please let me know if you have any questions and use the discussion board to bounce off ideas with your peers.

REVIEW CRITERIA

1. Significance: Is the importance of the proposed topic well-stated?
2. Background: Does the literature review clearly identify the problem-to-be-solved?
3. Innovation: Does the solution presented appear innovative based on what is outlined as background?
4. Approach: Do the proposed experiments logically address the problem-to-be-solved?
5. Expected Outcome & Future Directions: Are the expected outcomes of the project clearly stated? Are the future directions/broader impact of the proposed research described?
6. Format: Are the page limits and font size specifications respected?
7. Clarity: Is the proposal easy to read and understand?

Comments (Please describe the overall strengths and weaknesses of this proposal in 50-100 words)

Assignment 4: Peer Review

I received your first proposals. Two of your classmates, as well as myself, will critique the proposals. All proposals are available on Canvas under "Proposals for Peer Review" folder. You are assigned to critique the proposals from the two students posted as a custom comment under "Peer Review" assignment and were also emailed to you. Enter your comments and scores for the proposals at the following [LINK TO GOOGLE FORMS]. Use the drop-down menu to pick the name of the peer whose proposal you are reviewing. The proposal reviews need to be entered into the Google Form by [DATE].

Your proposal reviews will partition into your overall grade, which you will be scored for your thoroughness and critical reviewing.

Assignment 5: Revised Final Proposal

I uploaded the reviewer comments and scores for your proposal under “First Proposal” assignments on Canvas. The proposal and proposal critique grades are posted on Canvas.

The final proposal-related assignment is the following:

- Revise the proposal addressing the reviewer comments for your proposal (staying within the previously outlined page and formatting restrictions).
- Write a half-page (11 Arial, single-spaced, 1-inch margins all around) rebuttal to the reviewer comments indicating how you addressed them. Please refer to the sample proposal in Canvas under Reading/Proposal Related for the rebuttal language/style. 
- Upload your single electronic file (pdf document submission on Canvas Assignments) including the Rebuttal, Specific Aims, and Proposal by [DATE].

Assignment 6: Elevator Pitch

The presentations will be recorded to be reviewed by the instructor and the peers. The format is a **1 minute-long** "elevator pitch" for your proposal. Focus on the significance of the topic, the problem, your proposed solution, basic description of your approach, and what is the outcome/impact if successful. Being able to effectively communicate this information in 60 seconds is certainly challenging but a very important skill to acquire.

Use a single PowerPoint slide, record your presentation via Zoom Local Recordings as a mp4 file, and upload it on Canvas by [DATE]

Assignment 7: Elevator Pitch Peer Review

For this assignment, you will review the presentations uploaded under "Presentations for Peer Review" folder. You are free to watch all the presentations, but only required to provide comments/scores for the presentations from students whose proposals that you reviewed.

The presentation scores and comments are to be entered into the Google Forms at this link by [DATE].

Your presentation critiques will partition into your overall grade.

Proposal Peer Review Google Form

*The online Google Form collects the responses in a spreadsheet and allows for easily generating a “Summary Statement” for the students as the basis for formulating their “Response to Reviewers” and revising the proposal.*

| 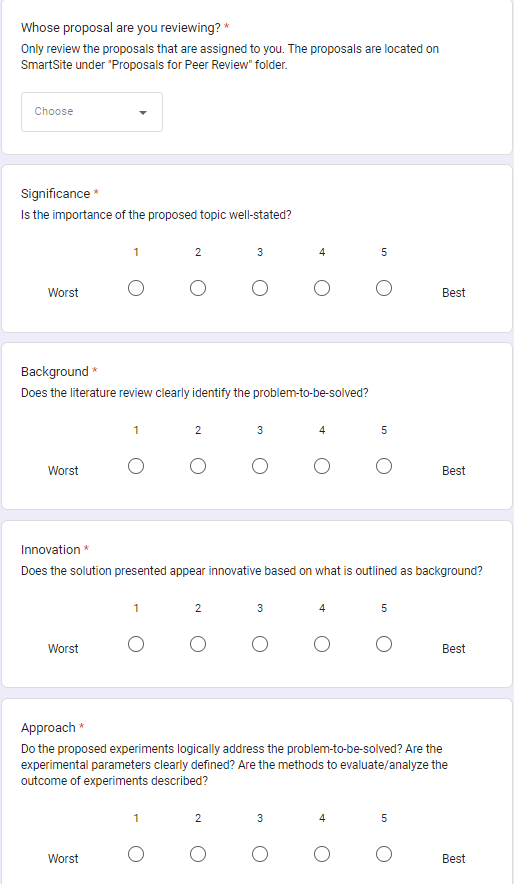 | 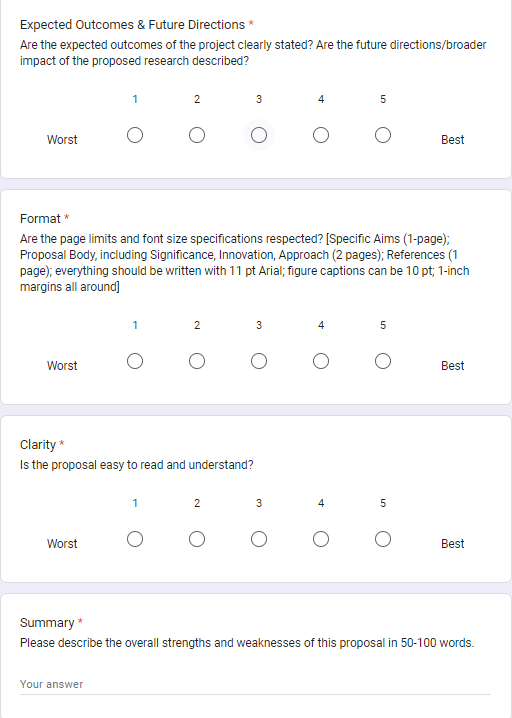 |
| --- | --- |

**Illustrative Readings**

*The readings (listed below) are uploaded onto Canvas course management platform and collated under specific topics throughout the course to complement the lectures.*

Introductory Topics

Voldman, J.; Gray, M. L.; Schmidt, M. A., Microfabrication in Biology and Medicine. *Annual Review of Biomedical Engineering* **1999**, 1 (1), 401-425*.*

Bhatia, S. N.; Ingber, D. E., Microfluidic Organs-on-Chips. *Nature Biotechnology* **2014**, 32 (8), 760-772

Seker, E.; Sung, J. H.; Shuler, M. L.; Yarmush, M. L., Solving Medical Problems with Biomems*. IEEE Pulse* **2011**, 2 (6), 51-59.

Surface Science

Introduction to Alkane Nomenclature (<http://masterorganicchemistry.com>)

Summary Sheet - Functional Groups (<http://masterorganicchemistry.com>)

Summary Sheet - Introduction to Chemical Reactivity, Nomenclature, Boiling Points, and Water Solubility (<http://masterorganicchemistry.com>)

Howgego, J., Rise of the Molecular Machines*. Education in Chemistry* **2012***,* 49 (5), 14*.*

Sperling, R. A.; Parak, W. J.*,* Surface Modification, Functionalization and Bioconjugation of Colloidal Inorganic Nanoparticles. *Philosophical Transactions of the Royal Society A: Mathematical, Physical and Engineering Sciences* **2010**, 368 (1915), 1333-1383.

Micro- and Nano-Fluidics

Unger, M. A.; Chou, H.-P.; Thorsen, T.; Scherer, A.; Quake, S. R., Monolithic Microfabricated Valves and Pumps by Multilayer Soft Lithography. *Science* **2000**, 288 (5463), 113-116.

Fredrickson, C. K.; Fan, Z. H., Macro-to-Micro Interfaces for Microfluidic Devices. *Lab on a Chip* **2004**, 4 (6), 526-533.

Squires, T. M.; Quake, S. R., Microfluidics: Fluid Physics at the Nanoliter Scale. *Reviews of Modern Physics* **2005**, 77 (3), 977.

Napoli, M.; Eijkel, J. C.; Pennathur, S., Nanofluidic Technology for Biomolecule Applications: A Critical Review. *Lab on a Chip* **2010**, 10 (8), 957-985.

Biology

Williams, D. F., On the Nature of Biomaterials. *Biomaterials* **2009**, 30 (30), 5897-5909.

Cell Culture Basics. Life technologies (Invitrogen) **2014**.

Kim, L.; Toh, Y.-C.; Voldman, J.; Yu, H., A Practical Guide to Microfluidic Perfusion Culture of Adherent Mammalian Cells. *Lab on a Chip* **2007**, 7 (6), 681-694.

Franz, S.; Rammelt, S.; Scharnweber, D.; Simon, J. C., Immune Responses to Implants–a Review of the Implications for the Design of Immunomodulatory Biomaterials. *Biomaterials* **2011**, 32 (28), 6692-6709.

An Introduction to Antibodies and Their Applications (EMD Millipore) **2013**.

Niemeyer, C. M., Nanoparticles, Proteins, and Nucleic Acids: Biotechnology Meets Materials Science. *Angewandte Chemie International Edition* **2001**, 40 (22), 4128-4158.

Fenno, L.; Yizhar, O.; Deisseroth, K., The Development and Application of Optogenetics. *Annual Review of Neuroscience* **2011**, 34, 389.

Berthier, E.; Young, E. W.; Beebe, D., Engineers Are from PDMS-Land, Biologists Are from Polystyrenia. *Lab on a Chip* **2012,** *12* (7), 1224-1237.

Medzhitov, R., The Spectrum of Inflammatory Responses. *Science* **2021**, 374 (6571), 1070-1075.

Brain Basics (NIH Educational Booklet)

The Chemistry of Health (NIH Educational Booklet)

Inside the Cell (NIH Educational Booklet)

The Structures of Life (NIH Educational Booklet)

Micro- and Nano-Fabrication

Shenderova, O.; Zhirnov, V.; Brenner, D., Carbon Nanostructures. *Critical Reviews in Solid State and Material Sciences* **2002**, 27 (3-4), 227-356.

Williams, K. R.; Muller, R. S., Etch Rates for Micromachining Processing. *Journal of Microelectromechanical Systems* **1996**, 5 (4), 256-269.

Williams, K. R.; Gupta, K.; Wasilik, M., Etch Rates for Micromachining Processing-Part II. *Journal of Microelectromechanical* Systems **2003**, 12 (6), 761-778.

Gates, B. D.; Xu, Q.; Stewart, M.; Ryan, D.; Willson, C. G.; Whitesides, G. M., New Approaches to Nanofabrication: Molding, Printing, and Other Techniques. *Chemical Reviews* **2005**, 105 (4), 1171-1196.

Chen, Y.; Pepin, A., Nanofabrication: Conventional and Nonconventional Methods. *Electrophoresis* **2001**, 22 (2), 187-207.
